# Spectral slowing is associated with working memory performance in children born very preterm

**DOI:** 10.1038/s41598-019-52219-0

**Published:** 2019-10-31

**Authors:** Julie Sato, Sarah I. Mossad, Simeon M. Wong, Benjamin A. E. Hunt, Benjamin T. Dunkley, Charline Urbain, Margot J. Taylor

**Affiliations:** 1 Department of Diagnostic Imaging, The Hospital for Sick Children, Toronto, Canada; 2 Department of Psychology, University of Toronto, Toronto, Canada; 3 Neuroscience & Mental Health Program, The Hospital for Sick Children Research Institute, Toronto, Canada; 4 Department of Medical Imaging, University of Toronto, Toronto, Canada; 5 UR2NF – Neuropsychology and Functional Neuroimaging Research Group at Center for Research in Cognition and Neurosciences (CRCN) and ULB Neurosciences Institute (UNI), Université Libre de Bruxelles (ULB), Brussels, Belgium; 6 Institute of Biomaterials and Biomedical Engineering, University of Toronto, Toronto, Canada

**Keywords:** Working memory, Cognitive neuroscience

## Abstract

Children born very preterm (VPT) often demonstrate selective difficulties in working memory (WM), which may underlie academic difficulties observed in this population. Despite this, few studies have investigated the functional networks underlying WM in young children born VPT, a period when cognitive deficits become apparent. Using magnetoencephalography, we examined the networks underlying the maintenance of visual information in 6-year-old VPT (*n* = 15) and full-term (FT; *n* = 20) children. Although task performance was similar, VPT children engaged different oscillatory mechanisms during WM maintenance. Within the FT group, we observed higher mean whole-brain connectivity in the alpha-band during the retention (i.e. maintenance) interval associated with correct compared to incorrect responses. VPT children showed reduced whole-brain alpha synchrony, and a different network organization with fewer connections. In the theta-band, VPT children demonstrated a slight increase in whole-brain connectivity during WM maintenance, and engaged similar network hubs as FT children in the alpha-band, including the left dorsolateral prefrontal cortex and superior temporal gyrus. These findings suggest that VPT children rely on the theta-band to support similar task performance. Altered oscillatory mechanisms may reflect a less mature pattern of functional recruitment underlying WM in VPT children, which may affect the processing in complex ecological situations.

## Introduction

Infants born preterm (<37 weeks gestational age [GA]) comprise approximately 10% of all live births globally^1^. This vulnerable population is a significant concern for families and healthcare systems due to their heightened risk of brain injury and poor neurodevelopmental outcomes. Although the degree of impairment varies, and some of these children do exceptionally well, about 30–60% of children born very preterm (VPT; <32 weeks GA) will experience significant cognitive and executive function difficulties, which can impact their success and integration at school^2,3^. Importantly, these cognitive difficulties appear to persist into adulthood, suggesting an altered trajectory of brain development in this population^4,5^. VPT infants are born during the third trimester of pregnancy, a critical period of brain development, which has given rise to theories about whether structural brain abnormalities reported in this population may trigger the functional reorganization of networks recruited for cognitive processes^6–8^. Importantly, VPT infants without structural brain injuries also experience impairments in cognition, attention, and executive function^9–13^, suggesting that cognitive difficulties may be an effect of functional brain injuries as a result of preterm birth. The negative impact of preterm birth on neurodevelopmental outcomes has been well documented, however little is known about the functional neural networks underlying these cognitive difficulties at early school-age.

Working memory (WM) is a core executive function linked with many higher-order cognitive abilities, and has been frequently reported as a difficulty in VPT compared to their full-term (FT) peers^14,15^. Impairments in WM impact various developmental disorders and underpin learning and academic difficulties^16–18^. WM deficits in preterm cohorts have been reported at early school-age^19–22^, and adolescence^3,21,23^, indicating selective vulnerability and disruption of the supporting structural and/or functional networks. One study noted that even VPT children without major neurological deficits and IQ within the normal range, showed impaired spatial WM performance compared to FT controls at 3–4 years of age^19^. Despite a large body of literature documenting WM difficulties in VPT children, several neuroimaging studies have found similar performance accuracy in preterm compared to FT children^24–26^. These studies highlight differences in functional neuroimaging studies with respect to task design, especially in children, as tasks are often simplified to increase task compliance and to attain enough trials for subsequent analyses. However, fMRI studies have shown that despite similar task performance, VPT children exhibit altered patterns of brain activity^24–26^. Thus, neuroimaging techniques may be more sensitive in detecting subtle group differences compared to behavioural measures, which lack the capacity to identify compensatory strategies or use of alternative neural networks to support similar performance.

In one study examining WM performance in 7 to 9-year-old VPT children, the authors found that while FT children recruited typical frontal activation, including the dorsolateral prefrontal cortex and the anterior cingulate cortex, VPT children displayed no frontal recruitment and reduced BOLD activity in the right parahippocampal area and left precuneus; likely reflecting less efficient neural processing^26^. Unlike these findings, another fMRI study found that VPT children (7–12 years) showed frontal activation during a visuospatial WM task, however, the pattern of frontal recruitment differed between VPT and FT children^25^. The authors found that VPT children showed reduced BOLD activity in the middle frontal gyrus and increased activity in bilateral superior frontal gyri compared to controls. Interestingly, the authors noted that functional differences were most pronounced in younger, low-performing VPT children^25^, whereas, older, high-performing VPT children recruited a similar network as FT children. These findings suggest that neural mechanisms supporting WM in younger VPT children show atypical network organization that shifts towards a more typical pattern of activation with increasing age. In contrast to previous studies, these findings support a “catch-up”, rather than an ongoing delay or persistent alterations in the WM network in VPT children.

To our knowledge, only one study has investigated the oscillatory dynamics underlying the maintenance of visual information in VPT children^8^. Neural oscillations can be characterized by their frequency, phase, and amplitude, by which functional interactions between discrete brain regions can be estimated. This approach allows for excellent spatial and temporal resolution to measure functional network interactions on a millisecond basis, as well as frequency-specific information on the neural underpinnings of WM^27–29^. It has been previously shown that reduced inter-regional phase synchrony in VPT children (7–8 years) during a visual short-term memory task was confined to the alpha-band (8 to 14 Hz), and was correlated with cognitive outcome^8^. The authors also noted that long-range phase synchronization in VPT children in the theta band (4 to 7 Hz) resembled the pattern observed in the alpha-band network in FT controls. The authors interpreted this to suggest that the alpha oscillatory mechanisms supporting WM processes may still be present in the VPT group but are shifted to a slower frequency band^8^. However, this study analyzed magnetoencephalography (MEG) data at only 19 sensors, thus important information regarding anatomical localization was not explored. Our previous work in 6-year-old FT children also supports the role of long-range alpha synchronization in mediating WM performance^30^. FT children showed a significant increase in mean whole-brain connectivity in the alpha-band during the retention period preceding subsequently correct compared to incorrect responses. Our network analyses revealed increased long-range alpha synchronization during WM retention, with dominant fronto-temporal connections, including the left dorsolateral prefrontal cortex, middle temporal and superior temporal gyri. Thus, alterations in long-range alpha synchronization may underlie selective WM difficulties observed in VPT children.

Different patterns of functional connectivity related to preterm birth, have been observed using both fMRI and MEG during infancy^31^, childhood^32–34^, and adolescence^7^. These studies have focused primarily on language network disruptions in the preterm brain. Thus, the present study addresses this gap in the literature regarding functional networks underlying visual WM processes in VPT children, as several studies have reported difficulties in this domain^8,15,19,20^. Selective difficulties in visual processing have also been reported in this population^35,36^, which can negatively impact visual WM processes. Further, similar versions of this visual WM task have been published in term-born children, both of which have reliably implicated long-range synchronization in multiple frequency bands, using MEG^30,37^. Together, these findings allow us a better understanding of the normal trajectory of visual WM maintenance, from which we can investigate alterations in the VPT brain.

We used MEG to investigate the functional network dynamics underlying WM maintenance in several frequency bands, including theta, alpha, beta and low gamma, in 6-year-old children born VPT compared to FT children. Visual WM maintenance, in the context of this paper, involves short-term memory processes and stores that maintain relevant visual information for short periods of time^38^. In a typical visual WM paradigm, a sample stimulus is presented (encoding), followed by a delay (retention), and then a test stimulus is presented requiring a response as to whether or not the test stimulus matches the sample (recognition). This type of WM task allows us to probe maintenance processes that occur during the delay period, when participants are actively holding visual information in WM. We expected that the frequency band important for mediating WM maintenance processes would show increased and sustained inter-regional synchrony during the retention interval associated with correct compared to incorrect responses, as shown in our previous work^30^. Other studies have also reported that performance declines when neural activity was not sustained during the retention period; thus, analyzing the connectivity patterns associated with correct and incorrect responses offers critical information about the neural substrates underlying successful WM performance^39,40^. Building on previous findings, we hypothesized that the VPT children would show reduced long-range alpha synchronization during the retention interval, compared to FT children^8^. We hypothesized that VPT children would recruit alternative networks in different frequency bands, such as the theta band, to support WM performance; and limited involvement from higher frequency bands such as beta and low-gamma. In addition, due to the protracted maturation of frontal lobes during early development, we expected reduced recruitment of prefrontal regions in VPT compared to FT children. In addition to between-group comparisons, functional networks within each group were investigated in frequency bands of interest.

## Results

### Demographic and behavioural data

The VPT and FT groups did not differ significantly in terms of age (*t*[33] = 1.37, *p* = 0.18) or sex distribution (χ^2^[1] = 0.04, *p* = 0.85), and were well-matched on maternal educational attainment (χ^2^[2] = 3.17, *p* = 0.21), which is an established proxy for socioeconomic status (see Table 1). Average gestational age at birth for our VPT group was 29.03 weeks (SD = 1.26). At the behavioural level, we found no significant difference between groups for the visual WM task performance inside the MEG scanner (*t*[33] = −0.10, *p* = 0.92; Table 1). Although we found no significant group difference for IQ (*t*[33] = 1.89, *p* = 0.07), or the digit and block recall of the WMTB-C (digit recall: *t*[33] = 1.46, *p* = 0.15; block recall: *t*[33] = 1.67, *p* = 0.10; Table 1), the VPT children performed more poorly than their FT peers.

**Table 1:** Demographic characteristics and behavioural results.

	FT group (N = 20)	VPT group (N = 15)
Age (years)	6.65 ± 0.34	6.48 ± 0.40
Sex (M: F)	10:10	8:7
**Maternal education level**		
Post-secondary training	2/20 (10%)	3/15 (20%)
University	12/20 (60%)	11/15 (73.3%)
Post-graduate training	6/20 (30%)	1/15 (6.7%)
Full-scale IQ	113.50 ± 14.16	105.27 ± 10.63
Visual WM accuracy (%)	80.15 ± 10.16	80.49 ± 9.01
**WMTB-C subtests** ** (std. score)**		
Digit Recall	113.20 ± 11.92	105.57 ± 9.72
Block Recall	107.79 ± 13.98	99.36 ± 14.46

Categorical variables were presented as frequency (percentage) and continuous variables as mean (standard deviation). WMTB-C = Working Memory Test Battery Second Edition. Std. score = standard score.

### Mean whole-brain connectivity timeseries: correct vs. incorrect responses

**Figure 1:**
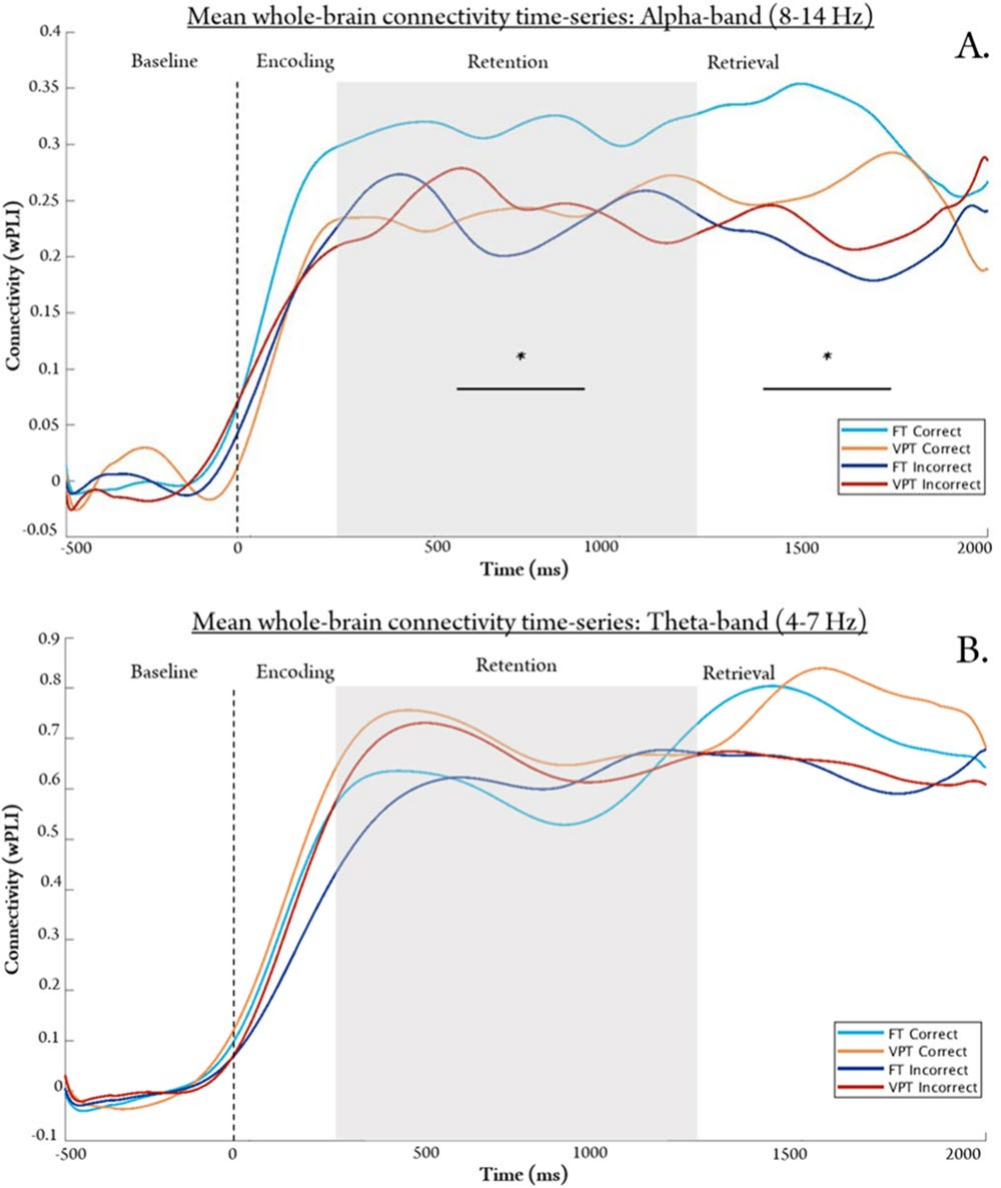
Mean whole brain connectivity in the alpha (**A**) and theta (**B**) bands during Baseline, Encoding, Retention and Retrieval phases. Time (in ms) is denoted on the x-axis, beginning at 500 ms before stimulus onset (0 ms). Connectivity values (weighted phase lag index [wPLI]) is shown on the y-axis. FT children displayed higher alpha connectivity during the retention interval preceding subsequently correct (light blue) compared to incorrect (dark blue) responses (*p*_*corr*_ = 0.001, represented by the *); VPT children showed no significant differences between correct and incorrect responses. No significant between/within-group differences were observed in the theta band, although the VPT group (in yellow/orange) had somewhat higher connectivity values during start of the retention period.

Figure 1 represents the connectivity timeseries used to determine if whole-brain networks during the retention (i.e. maintenance) period were differentially activated for correct and incorrect responses within and between groups. Unlike FT children who showed a significant increase in mean whole-brain alpha connectivity associated with correct compared to incorrect responses^30^, the VPT group showed no significant differences between correct and incorrect responses within the alpha frequency band (Fig. 1A; and see Supplemental Fig. 1 for plot with standard error bars). However, the alpha-band connectivity timeseries in the VPT group closely resembled that of the incorrect condition in the FT group. Compared to FT children, VPT children showed a slight but non-significant increase in whole-brain connectivity during the retention period in the theta-band (4–7 Hz; Fig. 1B; see Supplemental Fig. 2 for plot with standard error bars). Significant effects were not seen in beta or low gamma frequency bands (Supplementary Fig. 3). We therefore analysed the networks in the alpha and theta frequency bands.

### Within-group network analysis: Alpha-band (8–14 Hz)

**Figure 2:**
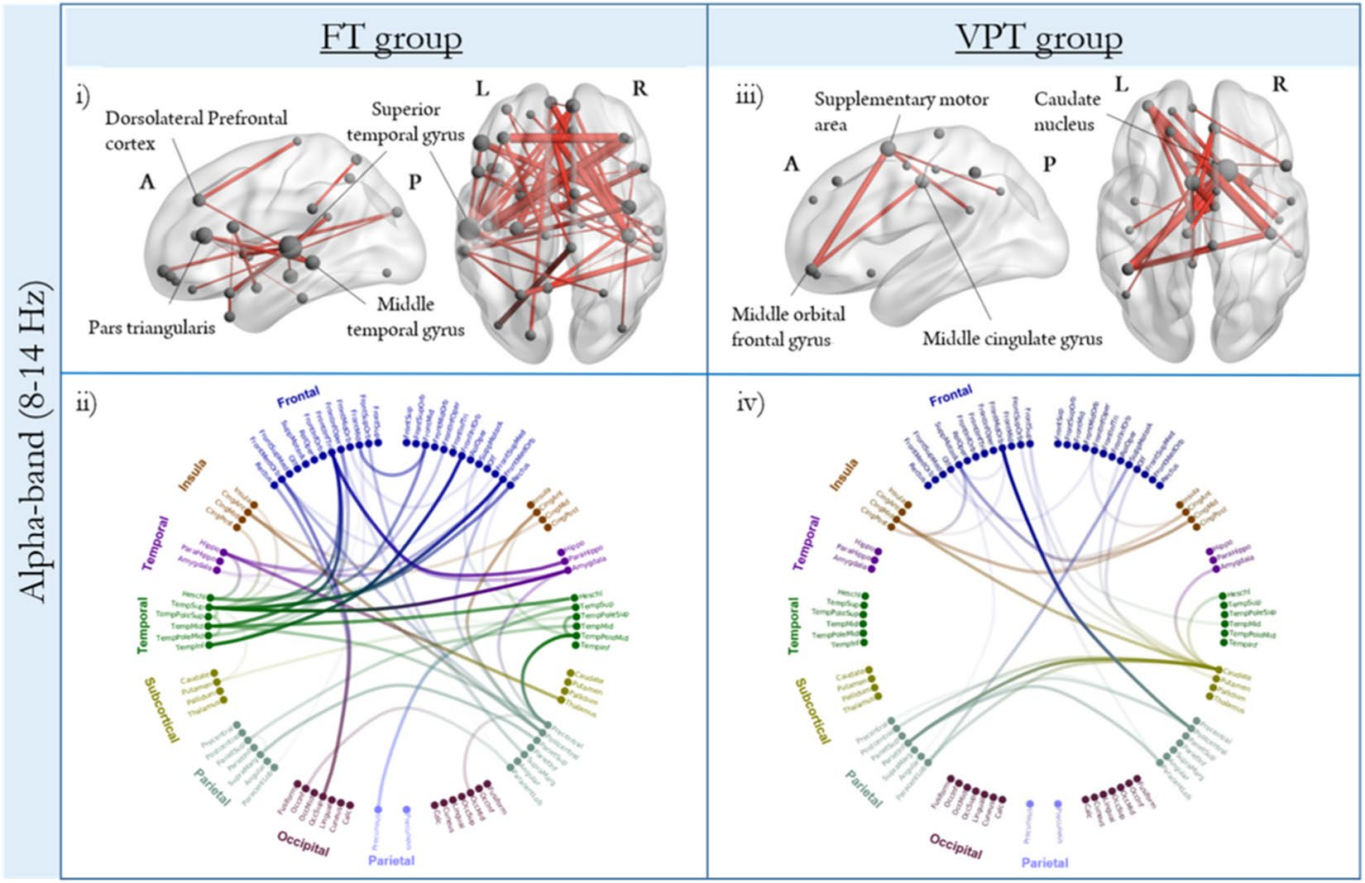
Within-group network analysis during the retention interval for both groups (*p*_*corr*_ = < 0.001) in the alpha-band (8–14 Hz). Functional connectivity (wPLI) was grand-averaged within each group and plotted on the glass brains (top row) and circle plots (bottom row). In the top row, significant regions (nodes) within the network are scaled by node degree, with larger spheres indicating nodes with a greater number of edges to other nodes in the network. In the bottom row, all significant edges are illustrated in the circle plots, with regions represented anatomically (left hemisphere nodes appear on the left-side of the plot). The opacity of the edges is scaled by connectivity strength, such that darker edges represent stronger connections between nodes.

FT and VPT children displayed elevated alpha synchronization during the retention interval (250–1250 ms) compared to baseline (−500 to 0 ms). However, the regional distribution of these networks varied between groups (Fig. 2; see Supplementary Table 1 for a complete list of network nodes). In the FT group, the alpha network included frontal, parietal and temporal regions (54 edges, 48 nodes; *p*_*corr*_ < 0.001). Central hubs in the network were identified using node degree, which reflects the degree of connectedness (i.e., the number of edges/connections a node has). Increased alpha connectivity was anchored in temporal regions, including the left middle temporal gyrus and superior temporal gyrus, left hippocampus, and the right middle temporal pole (Fig. 2i). In addition, the bilateral dorsolateral prefrontal cortex and the left pars triangularis also showed high degree within the network. The circular connectivity plots (Fig. 2 - bottom row) reflect all significant connections within each network, with all regions represented anatomically (left hemisphere nodes appear on the left-side of the plot). As illustrated by the circle plot, FT children also showed strong alpha synchronization between hemispheres, with dominant fronto-temporal connections (Fig. 2ii). The VPT group, however, showed quite a different network organization with fewer overall connections (Fig. 2iii,iv), and central hubs in the right caudate nucleus and the left Supplementary Motor Area (28 edges, 26 nodes; *p*_*corr*_ < 0.001).

### Within-group network analysis: Theta-band (4–7 Hz)

**Figure 3:**
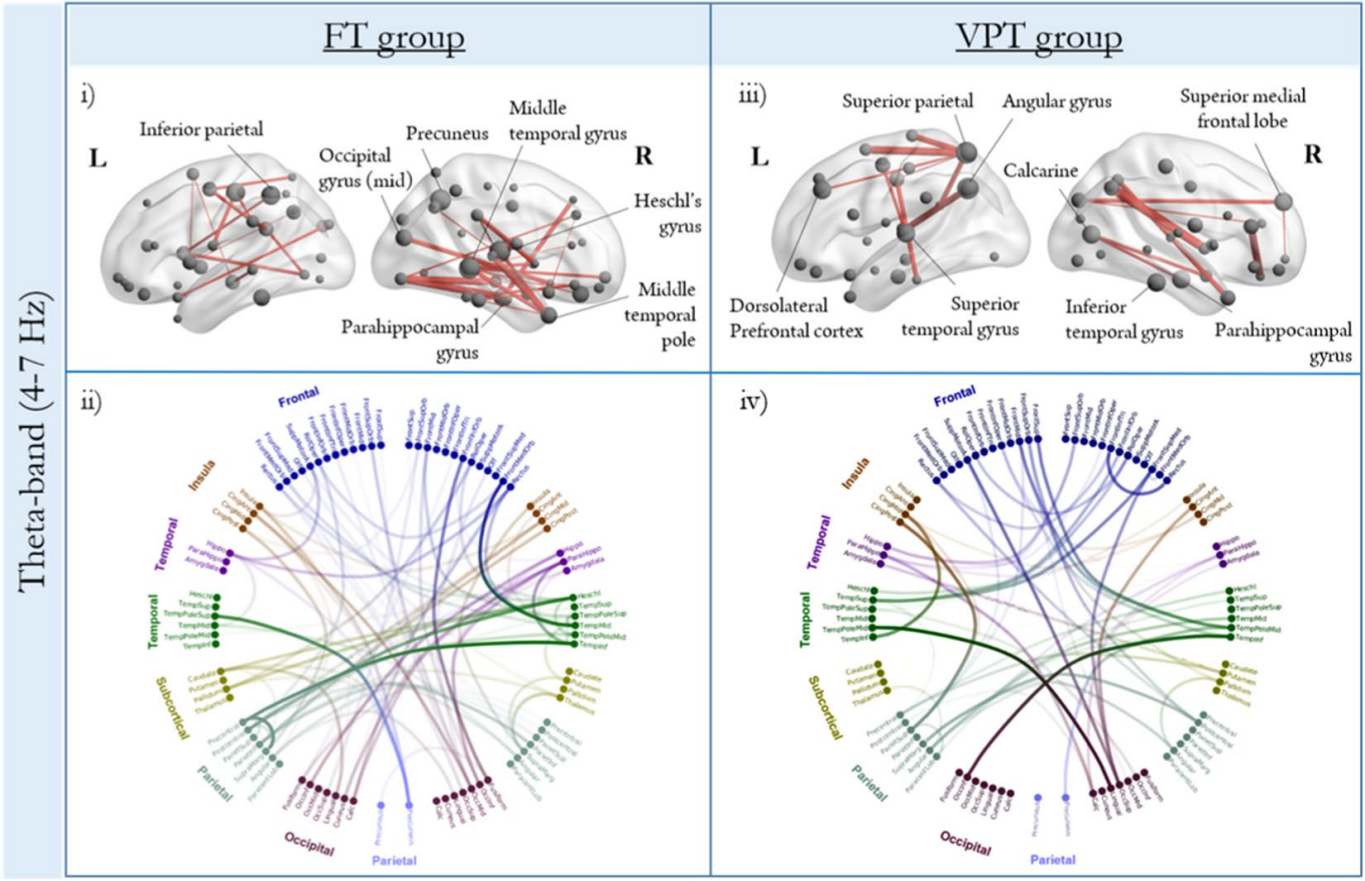
Within-group network analysis during the retention interval for both groups (*p*_*corr*_ = < 0.001) in the theta-band (4–7 Hz), plotted in the glass brains (top row) and circle plots (bottom row).

Elevated theta synchronization during the retention interval compared to baseline was observed for both groups (Fig. 3). The FT children (Fig. 3i) recruited a network anchored in temporal (right middle temporal, parahippocampal, right middle temporal pole, and Heschl’s gyri) and parietal (left parietal inferior lobe, left postcentral gyrus, and right precuneus) regions (95 edges, 67 nodes; *p*_*corr*_ = < 0.001; see Supplementary Table 1). Nodes with the highest degree within the theta-band network were right-lateralized in the middle temporal gyrus, Heschl’s gyrus and precuneus. In the FT group, right-lateralized temporal regions were connected strongly with left parietal and right frontal regions (Fig. 3ii). The VPT children also displayed a widespread network of temporal (left superior temporal, right inferior temporal and right parahippocampal gyri) and parietal (left angular gyrus, superior parietal) regions, as well as significant hubs in occipital areas, such as the right calcarine (59 edges, 54 nodes; *p*_*corr*_ = < 0.001; Fig. 3iii). Similar to FT alpha retention network, VPT children recruited left-lateralized hubs in the theta frequency, such as the left superior temporal gyrus and the dorsolateral prefrontal cortex. As illustrated by the circular connectivity plot, VPT children also showed strong bilateral temporo-occipital connections (Fig. 3iv).

### Between-group differences: mean network connectivity

**Figure 4:**
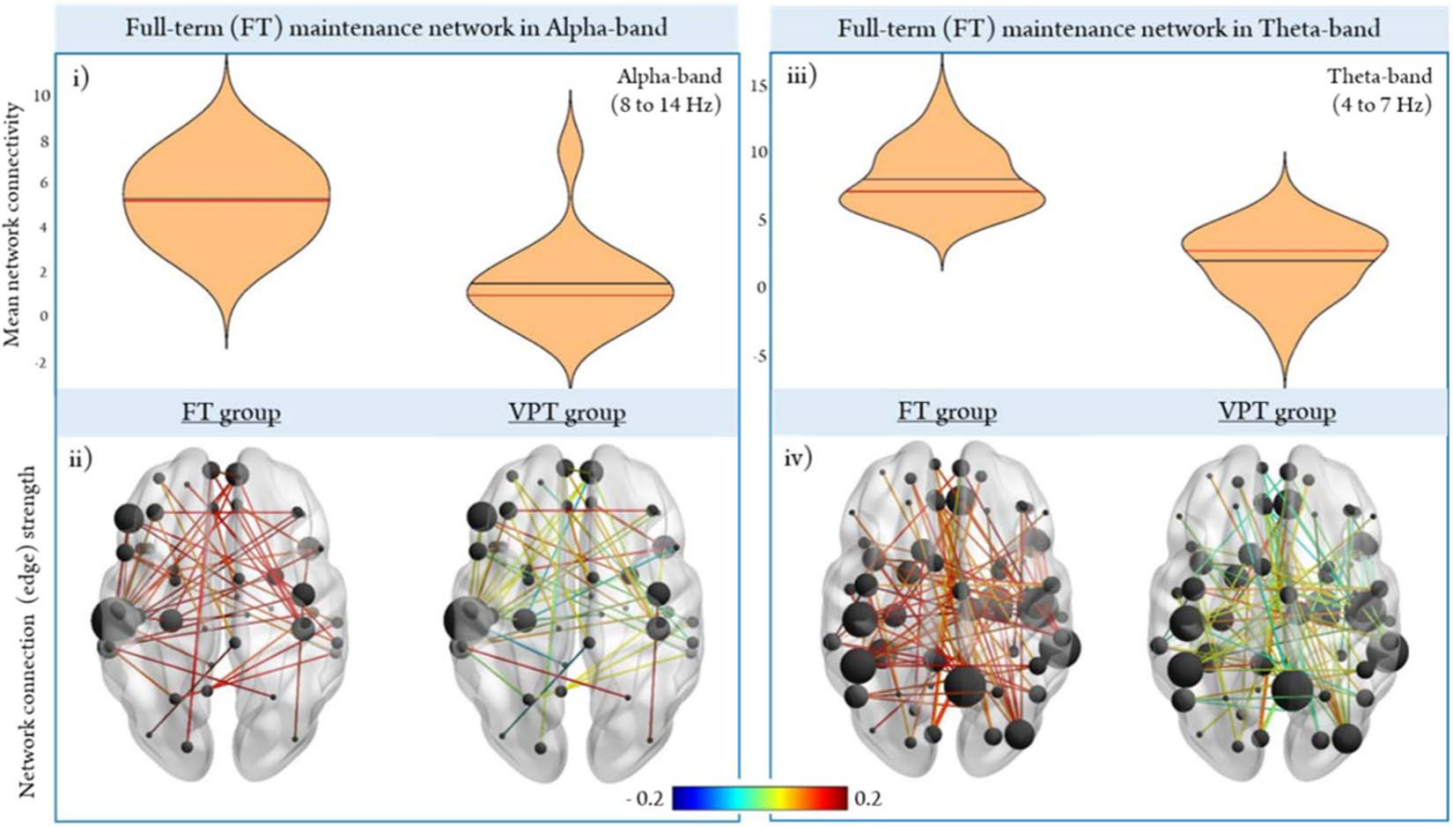
Between-group network analysis during the retention period. Significant connections recruited by the *FT group* in the alpha (**i,ii**) and theta (**iii,iv**) frequency bands were extracted and all other connections were masked for each subject to determine the extent to which these networks were recruited in each subject. Regional connection strength for each group are depicted in the lower panel (Alpha: ii; Theta: iv), with the colourmap scaled by strength (red is stronger, blue is weaker).

**Figure 5:**
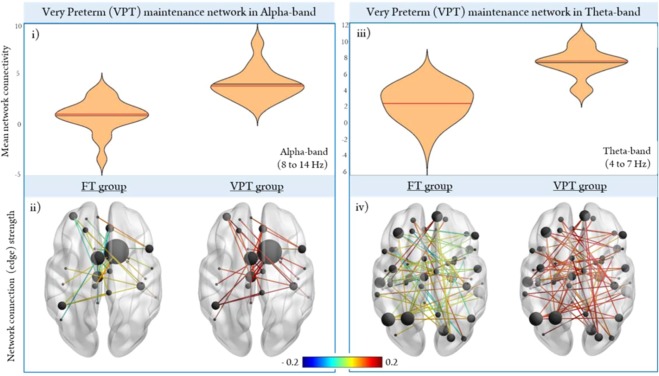
Significant networks exhibited during the retention period by the *VPT group* in the alpha (**i,ii**) and theta (**iii,iv**) bands were extracted and all other connections were masked to determine the extent to which these VPT networks were recruited in each subject.

Group differences at the *network level* were analyzed first using the alpha (54 edges, 48 nodes) and theta (95 edges, 67 nodes) networks identified in the FT group (Figs 2i and 3i, respectively). The network of regions recruited during the retention period in FT children was extracted to determine the extent to which VPT children activated this maintenance network. In the alpha-band, VPT children showed hypo-connectivity in the same network of nodes recruited during the retention period in FT children (*p*_*corr*_ < 0.001; Fig. 4i). Regional connection (edge) strength is plotted in the glass brains (Fig. 4ii) to provide a visual representation of the differential weights of the FT alpha-band network plotted in the VPT group. VPT children showed weaker connection strength in relation to the FT group, in nearly all connections within the FT alpha-band network. In the theta-band, we also found the same pattern of hypoconnectivity in the VPT group (*p* < 0.001; Fig. 4iii), showing weaker overall regional connections within the dense, widespread FT theta-band network (Fig. 4iv). These results reflect an under-utilization of this theta network activated by the FT and further support that VPT instead relied on another network to support task performance. We then tested the network recruited during the retention period in VPT children in both the alpha (28 edges, 26 nodes; Fig. 2ii) and theta (59 edges, 54 nodes; Fig. 3ii) frequency bands, against the FT group. The results in the both the alpha (Fig. 5i,ii) and theta-bands (Fig. 5iii,iv) show that FT children did not strongly engage either of the networks recruited by the VPT group during the retention interval. As depicted in the glass brains (Alpha: Fig. 5ii; Theta: Fig. 5iv), FT children demonstrated a pattern of overall weaker regional connections within the networks recruited by VPT children in both the alpha- and theta-bands. Thus, between-group findings show that both groups recruit distinct networks in the alpha and theta-bands to support task performance.

### Brain-behaviour associations

**Figure 6:**
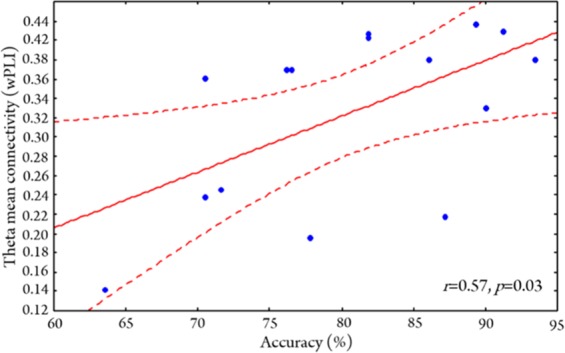
Pearson correlations between theta whole-brain connectivity (wPLI) and WM performance accuracy in the *VPT group* revealed a significant, positive association (*r* = 0.57, *p* = 0.03).

Pearson’s correlations were used to determine the association between mean whole-brain connectivity and WM task performance (% accuracy) in VPT and FT children. We averaged whole-brain connectivity over the retention interval (normalized to baseline) for correct trials only. In our previous work, we established a positive, but non-significant association between mean alpha connectivity and task performance in FT children^30^. This association was also not significant in the VPT group. Mean whole-brain connectivity in the theta-band, however, was positively associated with task performance in the VPT group (*r* = 0.57, *p* = 0.03; Fig. 6), while it was not in the FT group (r = −0.13, p = 0.59; see Supplemental Fig. 4). Gestational age within the VPT group was not correlated with mean whole-brain connectivity or WM task performance (p > 0.05).

## Discussion

In the present study, we demonstrated that VPT children exhibit alternative networks in the theta-band to support similar task performance and recruit many similar network hubs found in the FT maintenance network in alpha. These findings demonstrate the oscillatory mechanisms supporting visual WM in VPT children are shifted to the slower theta-band, compared to the inter-regional alpha synchronization observed in the FT group. We also extend previous MEG findings conducted at the sensor-level, and demonstrate the specific anatomical regions recruited by VPT children during WM retention period, including the left dorsolateral prefrontal cortex, superior temporal gyrus, and superior parietal lobe^8^. At the behavioural level, we found VPT children performed more poorly than FT controls on all measures, with a clinically meaningful difference in IQ score (8-points lower). Although these differences were not significantly different between groups, this is consistent with a large literature that shows weaker performance on a range of cognitive tasks and in IQ in VPT children. Our VPT sample were in fact quite high performing, compared to other groups reported in the literature, and although not significant, these slightly lower IQ scores may impact academic achievement in this VPT sample.

At the whole-brain level, VPT children showed no significant difference between correct and incorrect responses during the retention interval in either the alpha or theta frequency bands; unlike the FT group who showed a significant difference in the alpha-band. Our previous report in healthy six-year-old FT children, revealed the important role of long-range alpha synchronization to support successful WM performance^30^. The functional role of alpha oscillations in visual WM is well-established in adult studies, showing an increase in alpha synchrony with increasing memory load among a specialized network of regions^27^. In the alpha-band, we also found that the pattern of connectivity observed in the VPT group closely resembled the connectivity timeseries of the incorrect condition in the FTs. While these results were averaged over the whole brain, which may reduce sensitivity to smaller effects driven by specific networks or regional hubs, differences between successful and unsuccessful responses were still observed in the FT group. Our results are in line with Doesburg *et al*. (2011) who also found reduced long-range alpha synchronization in VPT children, compared to FT controls^8^. These differences in functional connectivity during successful WM performance are important given our sample consisted of a healthy and high-performing group of VPT children who were well-matched with FT children for task performance, full-scale IQ, and maternal education. Thus, MEG functional connectivity analyses may serve as a more sensitive method of capturing subtle group differences underlying WM development that may otherwise be missed in behavioural or fMRI studies.

Network-based analyses in the FT group revealed a distributed network underlying successful WM maintenance in the alpha-band, including the left dorsolateral prefrontal cortex, superior and middle temporal gyri and other canonical language regions such as the left pars triangularis, suggesting that they may rely on sub-vocal rehearsal strategies to guide their performance^30^. Within-group network analysis in the VPT group showed a different network organization in alpha, with fewer overall connections than seen in the FTs. Further, unlike many of the network hubs found in the FT network that have been widely implicated in WM maintenance, VPT children had network hubs in the right caudate nucleus and Supplementary Motor Area. While both these regions have been implicated in the broader WM network, memory and learning, and goal-directed behaviour^41,42^, the specific functional role of either has not been elaborated. The recruitment of these regions without the classic fronto-parietal involvement, may reflect compensatory strategies to aid in WM performance and/or preparation of a motor response (i.e., button press)^43^. Significant between-group differences in the alpha-band confirmed that VPT children showed reduced connectivity in the same network of regions recruited during WM maintenance by the FT group, and instead showed higher connectivity in an alternative network in alpha.

Further analyses at the whole-brain level, revealed that VPT children showed a positive trend towards increased connectivity in theta during the retention interval compared to the FT group. Doesburg *et al*. (2011) reported a significant peak in the theta frequency (~6 Hz) in VPT children, suggesting a potential slowing of the alpha oscillatory mechanisms observed in FT children^8^. They later showed that during a passive viewing task, slowing of alpha oscillations in VPT children involved prefrontal cortical regions^44^. Children born VPT show an altered and/or delayed developmental trajectory of executive processes, many of which depend on the maturation of frontal regions and reciprocal connections with other cortical and sub-cortical structures^45^. Thus, a disturbance of frontal lobe maturation during the preterm period may trigger a reorganization of functional networks recruited for WM, resulting in altered functional connectivity during WM maintenance involving the slower frequency band of theta. Further, the maturation of alpha oscillatory mechanism important for WM may be particularly susceptible to disruption following early life adversity. Due to the cross-sectional nature of these analyses, it is unclear whether these differences represent a functional reorganization of networks or a delay in alpha oscillatory mechanisms supporting WM. The latter explanation is supported by previous work suggesting a gradual shift from lower to higher frequencies across development^46,47^.

Within-group network analysis in the theta frequency showed that FT children recruit a widely distributed network including temporal, parietal, occipital, and frontal regions; this is in contrast to their smaller alpha network, with hubs lateralized to the left hemisphere. The VPT network in theta, also comprised prefrontal, parietal, occipital and temporal connections. Within-group network findings suggested that VPT children relied on this theta network to support task performance, as they were engaging similar network hubs (left dorsolateral prefrontal cortex and superior temporal gyrus) to those found in the FT control network in the alpha band, including the pars opercularis and pars triangularis. This suggests that VPT children, similar to FT controls, may also be engaging in subvocal strategies to guide their performance. In addition, the VPT theta network also included the left superior parietal cortex as hub region, which has been implicated in executive aspects of WM^48,49^. In adult studies, the superior parietal cortex appears to be more right-lateralized during spatial WM tasks^49^; thus, with development, lateralization may shift to the right hemisphere as maintenance operations continue to mature. Significant between-group differences showed that VPT children do not strongly engage the same network of regions activated during WM retention by FT children in theta. The VPT group instead rely on a distinct network in theta, showing significantly higher connectivity compared to the FTs. Together with the within-group network findings, our results demonstrate that VPT children engage an alternative network in the theta-band to support task performance. Supporting this explanation, our brain-behaviour correlations revealed a positive relation between mean whole-brain theta connectivity during the retention interval and task performance only in the VPT group.

Despite similar behavioural performance, we observed altered patterns of functional connectivity in VPT children, who demonstrated a frequency shift involving the theta band to support task performance. Our findings suggest that that early life adversity sustained in children born VPT may disrupt the maturation of alpha oscillatory mechanisms involved in WM maintenance, as seen in age-matched FT children. Importantly, these differences were not due to performance effects or intellectual disability, as VPT children included in these analyses were healthy and high-performing. Higher theta connectivity during task engagement may also serve as a marker of resiliency in children born VPT, as this was associated with improved behavioural performance. Further research is needed to investigate whether VPT children display patterns of functional connectivity similar to FT children with increasing age, or whether these altered network mechanisms reflect persistent differences between VPT and FT children.

## Methods

### Participants

All children were imaged at 6 years of age at the Hospital for Sick Children (SickKids). Participants were selected from a cohort of 52 children (24 VPT: 28 FT); some of whom were excluded based on poor task performance (8 VPT: 6 FT), and excessive movement in the MEG scanner (1VPT: 2 FT). Thus, the study groups included 15 children born VPT (mean age at scan 6.48 years [SD = 0.4]; mean GA: 29.0 [SD = 1.3]) and 20 FT controls (mean age at scan 6.65 [SD = 0.3]; Table 1). The FT sample and related methods has previously been published^30^. Exclusion criteria included a current diagnosis or history of neurological or psychiatric disorder, uncorrected hearing or visual impairments. Children were also screened and approved for MEG and MRI compatibility (i.e., being without any metal implants or devices). VPT subjects were recruited as part of larger longitudinal study, conducted between 2009 and 2018 at SickKids hospital, and FT children were recruited through advertisements posted in-hospital, and flyers posted in local schools, or through word-of-mouth. All children provided verbal assent and parents gave written informed consent. This study was carried out in accordance with the recommendations of the Research Ethics Board at SickKids, who reviewed and approved the study protocol.

### Perinatal clinical and radiological measures

Perinatal clinical data was obtained by chart review during primary hospitalization in the neonatal intensive care unit. Information such as GA, birthweight, and sex for children born VPT are summarized in Table 2. Additionally, significant events during pregnancy (i.e., intrauterine growth restriction), measures of illness (i.e., APGAR score at 5 min, the Clinical Risk Index for Babies II [CRIB II], necrotizing enterocolitis, and sepsis) and presence of white matter injury are described in Table 2. Paediatric neuroradiologists and neurologists reviewed each of the structural T1- and T2-images for children born VPT shortly following birth. The grade of germinal matrix/intraventricular hemorrhage (GMH/IVH) was determined based on the Papile scale (0 to 4)^50^. T1-, T2-weighted and FLAIR images were also assessed at six years of age by paediatric neuroradiologists, and incidental findings were noted.

**Table 2:** Clinical and radiological characteristics at VPT birth.

Characteristic	Mean (SD) or number (%)*
Gestational age (weeks)	29.0 (1.3)
Birthweight (g)	1212.3 (243.9)
Males	8 (53.3)*
Intrauterine growth restriction	3 (20)*
APGAR score at 5 min	7.8 (1.4)
CRIB II	6.8 (2.3)
Sepsis (cultures positive)	4 (26.7)*
Necrotizing enterocolitis (stage 2 & 3)	0 (0)*
Bronchopulmonary dysplasia	2 (13.3)*
GMH (Grade 1–2)	2 (13.3)*
GMH (Grade 3–4)	4 (26.7)*
White Matter lesions	6 (40)*

*Characteristics reported as numbers (%) are indicated with an asterisk.

### Task and assessments

**Figure 7:**
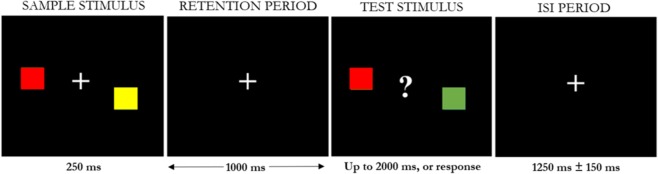
Stimuli for the visual WM task. Children were presented with the sample stimulus for 250 ms, followed by a 1000 ms retention interval. A second, test stimulus was then presented for up to 2000 ms, or until the child responded, with a question mark to prompt the child to respond. The inter-stimulus interval (ISI) period varied between 1100 and 1400 ms.

A detailed description of the visual WM task has been previously published^30^. Briefly, children were presented with a sample stimulus for 0.25 s, followed by a 1 s retention interval. A second test stimulus was then presented for up to 2 s, or until the child responded. The inter-stimulus interval (ISI) period, jittered between 1.1 and 1.4 s, followed the presentation of the test stimulus (Fig. 7). Children responded by pressing either a left or right button on a MEG-compatible button pad to indicate if the colours of the squares in the test stimulus were the same or different than the sample stimulus. In half of the trials the colour of one square changed between the sample and the test stimulus. The task was run for two blocks of 7 minutes, with a short break in between. All children received instructions and completed practice trials before MEG scanning to ensure understanding of the task.

Neuropsychological measures were used to assess cognitive ability in children. Children were administered the vocabulary and matrix reasoning subtests of the Wechsler Abbreviated Scale of Intelligence, Second Edition (WASI-II)^51^ to estimate IQ. WM ability was also assessed outside the scanner using the forward digit and block recall subtests of the Working Memory Test Battery for Children (WMTB-C)^52^, which are designed to engage the phonological loop and visuospatial functions of WM, respectively.

### MEG and MRI data acquisition, and source reconstruction

MEG data were recorded continuously (600 Hz sampling rate, 150 Hz anti-aliasing, and 3^rd^ order spatial gradient noise cancellation) while children lay supine in a magnetically shielded room, with a 151 channel CTF system (Coquitlam, Canada). Stimuli were presented using *Presentation* 20.0 software (Neurobehavioural Systems Inc., Albany, CA). Stimuli were projected into the magnetically shielded room via system of mirrors, and back-projected onto a screen positioned approximately 80 cm from the child’s eyes. The visual angle of the stimuli was 6.9°. Response timing and accuracy were recorded for each trial. Three fiducial coils were placed on the right and left pre-auricular points and the nasion to determine head position and movement in the MEG helmet and allow for continuous head motion correction. Before MRI recording, radio-opaque markers were placed in the same location as the MEG localization coils to co-register each participant’s MEG data with their anatomical MRI. T1-weighted MR images were acquired using a 12-channel head and neck coil (3T MAGNETOM Tim Trio, Siemens AG, Erlangen, Germany) at SickKids, with a 3D sagittal MRPAGE sequence: GRAPPA = 2, FA = 9°, TR/TE = 2300/2.96 ms, FOV = 28.8 × 19.2 cm, 240 × 256 matrix, 192 slices, slice thickness = 1.0 mm, scan time = 5:03 min.

Our analyses focused on the 1 s retention period; data were processed and analyzed using MATLAB R2017b software (Mathworks Inc., Natick, MA) and the FieldTrip toolbox (20150908 release)^53^. Data epochs for retention periods associated with correct and incorrect WM responses were separated for analyses, and epoched −1 s pre-stimulus to 2 s post-stimulus onset (i.e., the presentation of the sample stimulus). The large initial epoch allows for truncation post-filtering to minimize boundary effects at the beginning and end of these data epochs. For incorrect trials, only false alarm trials in which a response was provided (i.e., button press) were included in the analyses (miss trials were excluded). MEG data were then band-passed filtered from 1 to 150 Hz, with a 4^th^ order two-pass Butterworth filter and a 60 Hz notch filter. Following application of ICA, trials were rejected from analysis if greater than 10% of samples in any given trial exceeded 5 mm from the initial median head position or if the MEG signal from sensors was greater than 2000 fT. This is consistent with head motion thresholds reported in other developmental MEG studies^26,54,55^. The ICA function in FieldTrip^53^ was used to remove artefacts related to cardiac activity or ocular movements. Artefactual ICA components were identified visually and excluded from MEG data. The 90 regions of the Automated Anatomical Labelling (AAL) atlas^56^ were unwarped from the MNI template brain space into corresponding locations for each child’s headspace. MEG timeseries data for each of the 90 AAL regions were estimated using a Linearly Constrained Minimum Variance beamformer^57^. Beamformers act as spatial filters, suppressing signal and noise from sources of no interest, to estimate activity at each source of interest in the brain. The beamformer was run with 5% regularization and covariance matrices calculated on processed epochs.

### Functional connectivity analysis

The estimated timeseries for each AAL region were then filtered into frequency bands: theta (4–7 Hz), alpha (8–14 Hz), beta (15–30 Hz) and low gamma (30–55 Hz). A Hilbert transform was applied to the filtered timeseries to extract instantaneous phase at each time point and frequency band. Phase synchrony was calculated using the weighted phase lag index (wPLI) between all pairwise combinations of the AAL atlas, resulting in a 90 × 90 connectivity matrix for each subject, at each time point. The wPLI quantifies the phase synchrony of two sources by calculating the asymmetry of the distribution of the instantaneous phase differences between the timeseries of the sources. The wPLI provides values ranging from 0 (no phase locking, random phase difference) to 1 (maximum phase locking, constant phase difference); and is based on the magnitude of the imaginary component of the cross-spectrum^58^.

Connectivity timeseries within each frequency band and condition (i.e., correct vs. incorrect responses) were calculated for both groups to determine frequency bands of interest. The average connectivity timeseries data further allowed us to examine how whole-brain networks were differentially activated during successful and unsuccessful maintenance of visual stimuli. The values were z-scored to the baseline (between −0.5 and 0 s), then averaged across subjects within group, resulting in a single timeseries for each condition. A permutation test (*n* = 1000) was employed to identify significant differences between the connectivity timeseries of each condition, at each time point. On each of the 1000 permutations, the condition labels of each timeseries were randomly permuted, the t-statistic computed between the randomly labelled timeseries, then thresholded at t >1.7. The length of the largest contiguous supra-threshold window was recorded into the null distribution. Windows of contiguously supra-threshold differences in the unpermuted timeseries were therefore statistically significant and corrected for multiple-comparisons, if supra-threshold windows were larger than the 95^th^ percentile of randomly obtained clusters.

To determine the network of regions engaged in successful WM maintenance (i.e., correct hits), connectivity matrices were compiled and submitted to a Network-Based Statistic (NBS)^59^. Statistical contrasts between conditions of interest (retention vs. baseline period) were computed within each frequency band and for each group separately. The retention period reflected the 1-second interval between the presentation of the sample and test stimulus, and the baseline period was the pre-stimulus ISI period. NBS identifies network components (i.e., contiguous set of inter-regional connections) that significantly differ between conditions of interest. NBS controls for the Family-Wise Error Rate (FWER) when performing mass univariate testing on a graph and assigns statistical significance at the level of the network, rather than at the level of each pairwise connection. The network components are ascribed a corrected p-value by the FWER using permutation testing (*n* = 5000). Conservative thresholds (t > 2.0) were chosen and adapted to the data distribution under investigation^59,60^. Node degree, which is the number of edges a node has, was used to identify brain regions that were network hubs.

Between-group statistics were calculated using the sum of network connectivity, which takes the sum of the wPLI (z-scored to the baseline) value for all connections in one network (within each frequency band) and masking all other connections for each subject, which determines the magnitude to which this network is recruited in each subject. This analysis allowed us to determine the extent to which each participant activates this specific network, resulting in a single value per subject. Significant group differences were tested using a permutation test (*n* = 5000). As we were also interested in the associations between brain and behaviour, we averaged wPLI values over the 1-second retention interval (normalized to baseline) within each frequency band of interest, and correlated them with WM behavioural accuracy. Associations were tested for each group.

## Supplementary information

Supplementary Materials

## Data Availability

The datasets analysed during the current study are available from the corresponding author upon reasonable request.
