# Supplementary material for: Spectral slowing is associated with working memory performance in children born very preterm
Source: Sci Rep. 2019 Oct 31;9:15757. doi: 10.1038/s41598-019-52219-0 (PMC6823447; doi:10.1038/s41598-019-52219-0)
Supplement: Supplementary file 1 — Supplementary Materials [file 41598_2019_52219_MOESM1_ESM.pdf]

Spectral slowing is associated with working memory performance in children born very preterm

*Julie Sato[1,2,3], Sarah I. Mossad[1,2,3], Simeon M. Wong[3,6], Benjamin A.E. Hunt[1,3], Benjamin T. Dunkley[1,3,4], Charline Urbain[5], Margot J. Taylor[1,2,3,4]

[1] Department of Diagnostic Imaging, The Hospital for Sick Children, Toronto, Canada

[2] Department of Psychology, University of Toronto, Toronto, Canada

[3] Neuroscience & Mental Health Program, The Hospital for Sick Children Research Institute, Toronto, Canada

[4] Department of Medical Imaging, University of Toronto, Toronto, Canada

[5] UR2NF – Neuropsychology and Functional Neuroimaging Research Group at Center for Research in Cognition and Neurosciences (CRCN) and ULB Neurosciences Institute (UNI), Université Libre de Bruxelles (ULB), Brussels, Belgium

[6] Institute of Biomaterials and Biomedical Engineering, University of Toronto, Toronto, Canada

*Corresponding author:

Julie Sato, MA
Diagnostic Imaging, Hospital for Sick Children
555 University Avenue
Toronto, ON Canada M5G 1X8
Tel: 416-813-7654 ext. 309118
Email: julie.sato@sickkids.ca

Supplementary Materials

Supplementary Table 1. Visual WM maintenance networks in theta and alpha frequency bands in very preterm and full-term children.

		**Theta-band (4-7 Hz)**		**Alpha-band (8-14 Hz)**	
AAL region	Hemisphere	FT group	VPT group	FT group	VPT group
Precentral	L	4	3		1
	R	1		4	
Frontal Sup.	L	1	2		2
	R	1			
Frontal Sup. Orb.	L	2	2	1	1
	R	2	2		
Frontal Mid (dlPFC)	L		4	3	
	R	2		2	
Frontal Mid Orb.	L	1		2	3
	R				
Pars Opercularis	L		2	3	
	R		1	1	3
Pars Triangularis	L	1		5	
	R	1	3		
Frontal Inf. Orb.	L				
	R	2	2	1	
Rolandic Oper.	L	2	1		
	R	3	3		1
Supp. Motor Area	L	3	2		5
	R			2	1
Olfactory	L	1			1
	R	1			2
Frontal Sup. Medial	L				1
	R		4	1	
Frontal Med Orb.	L	2		3	
	R	3	1	4	
Rectus	L	3	2	1	
	R	5	1	2	
Insula	L	4		1	
	R	1	1		
Cingulum Ant.	L	3		2	
	R	1	1	2	3
Cingulum Mid	L	-	3		4
	R	3	2	1	2
Cingulum Post	L	4	1	1	1
	R	1			
Hippocampus	L	4	2	4	
	R	3			
ParaHippocampal	L		3		
	R	6	4	1	
Amygdala	L		1	2	
	R	3	2	3	1
Calcarine	L	2			
	R		4		
Cuneus	L	3			
	R				
Lingual	L	2			
	R	1	1		

Occipital Sup.	L	2		2	
	R	3	3		
Occipital Mid	L				
	R	5	1	1	
Occipital Inf.	L	1	1	1	
	R	3	2		
Fusiform	L	5			
	R	2			
Postcentral	L	6	2		1
	R	1	3	4	3
Parietal Sup.	L	1	5	2	
	R	2		1	
Parietal Inf.	L	6			3
	R	3			
SupraMarginal	L	3	2	2	
	R	3		2	
Angular	L	4	5		1
	R	3	3		
Precuneus	L	1		2	
	R	7	2		
Paracentral Lobule	L		2	1	2
	R			2	2
Caudate	L	4			
	R				9
Putamen	L		1	2	
	R	2	2		1
Pallidum	L	4			
	R		2		
Thalamus	L		1		
	R	4		1	
Heschl	L		2	3	
	R	7	1	2	1
Temporal Sup.	L		4	8	
	R	2	1	2	
Temporal Pole Sup.	L	2	2	2	
	R	3		2	
Temporal Mid	L	2		4	
	R	7	1	2	1
Temporal Pole Mid	L	1	2	2	
	R	5	3	4	
Temporal Inf.	L		1	2	
	R	4	4		

All significant AAL brain regions (nodes) in each network are represented above ($p_{corr} > 0.001$), using node degree, which is the number of significant connections/edges a node has. Nodes with highest degree are considered network hubs and are therefore crucial to efficient network communication. The FT alpha-network is comprised of 48 nodes (first column), the VPT alpha-network 26 nodes (second column), the FT theta-network 67 nodes (third column), and the VPT theta-network 54 nodes (fourth column).

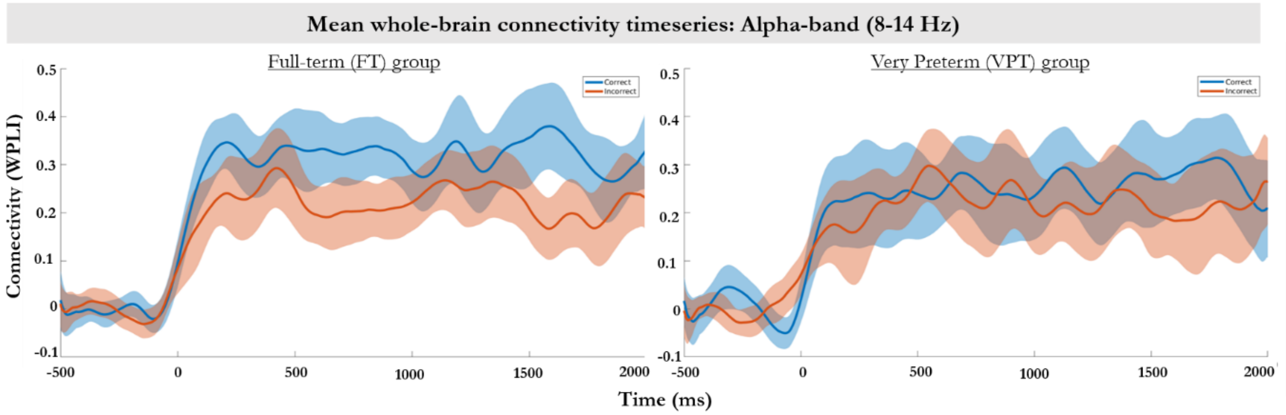

Supplementary Fig 1. Mean whole brain connectivity timeseries with standard error bars (blue and red shaded regions) for FT (left figure) and VPT (right figure) children in the alpha band (8-14 Hz). The blue line represents the correct condition, and the orange line is the incorrect condition.

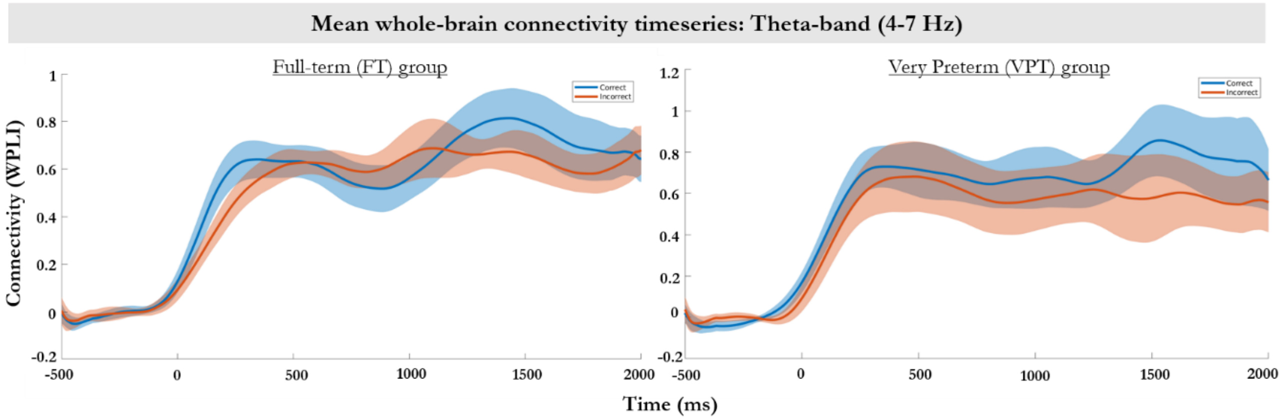

Supplementary Fig 2. Mean whole brain connectivity timeseries with standard error bars for FT (left figure) and VPT (right figure) children in the theta band (4-7 Hz). The blue line represents the correct condition, and the orange line is the incorrect condition.

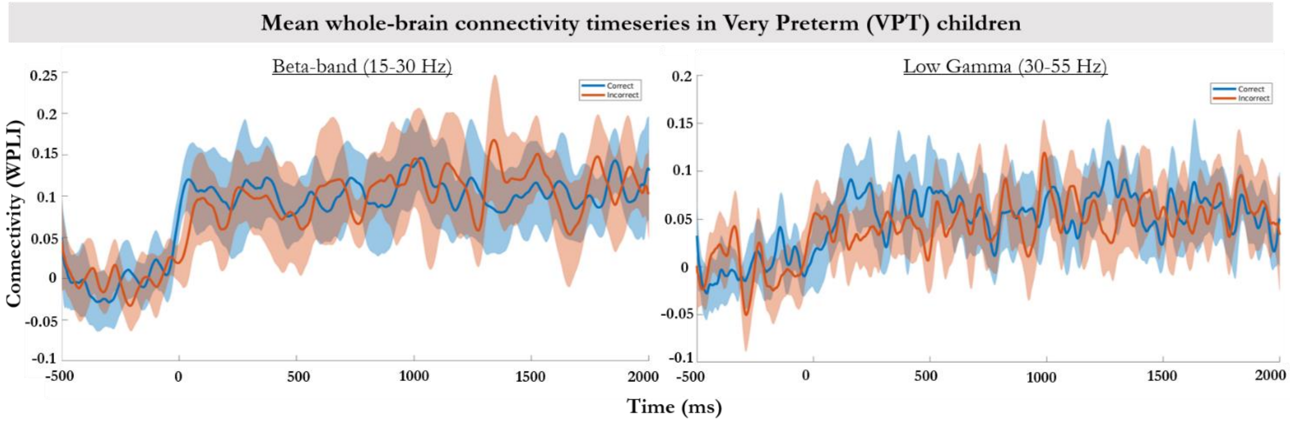

Supplementary Fig 3. Mean whole brain connectivity timeseries for VPT children in the beta (left figure) and low-gamma frequency bands (right figure). The blue line represents the correct condition, and the orange line is the incorrect condition.

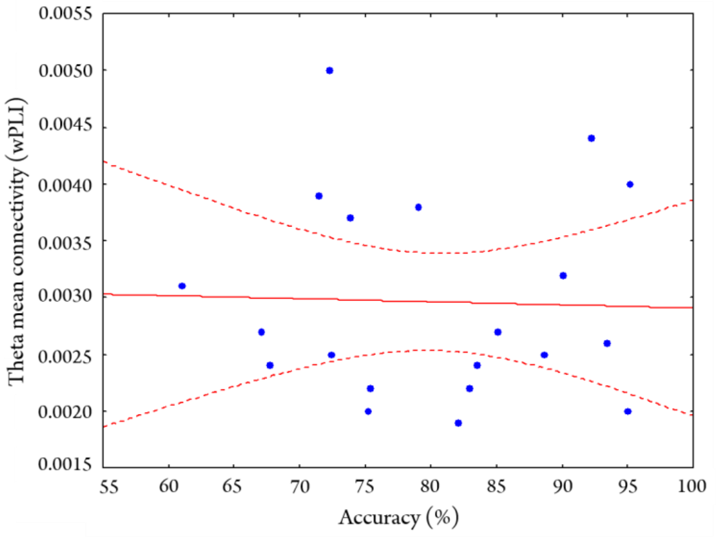

Supplementary Fig 4. Pearson correlations between theta whole-brain connectivity (wPLI) and working memory behavioural accuracy in the *FT group* was non-significant (r=-0.13, p=0.59).
